# First nationwide case – case study on factors associated with emerging methicillin-resistant *Staphylococcus aureus spa* type t4549 in Denmark, 2022–2023

**DOI:** 10.1017/S0950268825000299

**Published:** 2025-03-12

**Authors:** Johanna J. Young, Tjede Funk, Tinna Ravnholt Urth, Steen Ethelberg, Jesper Larsen, Andreas Petersen

**Affiliations:** 1 Department of Infectious Disease Epidemiology and Prevention, Statens Serum Institut, Copenhagen, Denmark; 2 ECDC Fellowship Programme, Field Epidemiology path (EPIET), European Centre for Disease Prevention and Control (ECDC), Stockholm, Sweden; 3 Department of Public Health, Global Health Section, University of Copenhagen, Copenhagen, Denmark; 4 Department of Bacteria, Parasites and Fungi, Statens Serum Institut, Copenhagen, Denmark

**Keywords:** Denmark, lower extremities, Methicillin – *Staphylococcus aureus* resistant to (MRSA), risk factors, swimming

## Abstract

Methicillin-resistant *Staphylococcus aureus* (MRSA) *spa* type t4549 is increasingly prevalent in Denmark, yet its epidemiological sources remain unclear. This study aimed to generate hypotheses about possible risk factors that may be associated with MRSA t4549 infections. We conducted a nationwide case – case questionnaire study comparing MRSA t4549 cases to other MRSA types (t002, t008, t127, t304, and t223) reported between January 2022 and November 2023. The analysis, which included descriptive statistics and logistic regression, found that 75% of MRSA t4549 cases were male. Infections were significantly more frequent in the foot (28%) and toe (54%) compared to other MRSA types. Key risk factors identified were contact with pheasants (OR = 8.70; 95%CI 1.25–174.29), participation in indoor team sports (OR = 7.54, 95%CI: 1.58–54.82) and swimming (OR = 4.15, 95%CI: 1.97–9.03). Although the limited number of cases warrants cautious interpretation, it is crucial to emphasize the need for preventive measures at both the individual and sports facility levels. Further environmental studies are needed to clarify the role of the environment and wildlife in MRSA t4549 transmission. The increasing prevalence of this *spa* type in Denmark underlines the importance of implementing effective public health strategies to reduce the risk of MRSA transmission.

## Background

Methicillin-resistant *Staphylococcus aureus* (MRSA) has become a leading cause of bacterial infections, and infections can be difficult to treat with existing antibiotics [1]. Mandatory laboratory and clinical notification of all cases of MRSA in Denmark has existed since November 2006. In Denmark, MRSA infections are notifiable, and all isolates are typed at the Statens Serum Institut (SSI). In 2022, nearly half of all new MRSA cases had no known risk factors for MRSA acquisition and were therefore defined as community-acquired with no known exposure [2]. One of the MRSA types classified in this category is MRSA with the *spa* type t4549 and multilocus sequence type ST630 (hereafter referred to as MRSA t4549). It is a Panton-Valentine leukocidin (PVL) negative MRSA strain that differs from other MRSA *spa* types. MRSA t4549 has been associated with food, animals, and humans in the past, but the epidemiological source of this type remains unknown [3]. Given that MRSA t4549 lacks the *scn* gene typical to human-associated strains, it is likely that the source or reservoir of this strain is an animal [4]. However, this remains to be confirmed. In addition, it also lacks the typical wall teichoic acids that enable other *S. aureus* clones to colonize the anterior nares [5]. All these differences from other MRSA strains make this MRSA *spa* type particularly interesting to study.

MRSA t4549 was first found in Denmark in 2012 in a traveler from Asia, where this lineage is endemic [6]. Since then, it has rapidly increased and is now one of the most commonly found MRSA *spa* types in the country [2], showing a widespread spatial pattern also in individuals without a travel history.

Since its introduction in Denmark, little is known about the epidemiology and source of this lineage. A better understanding of the factors associated with MRSA t4549 infection will not only increase knowledge but may also allow informing public health measures to help limit the spread of this type of MRSA infection.

This study therefore aimed to describe the epidemiology of MRSA t4549 cases and to identify factors associated with MRSA t4549 infection in order to generate hypotheses about possible risk factors that may be involved in infection with the emerging MRSA t4549 lineage and to give an indication of the natural reservoir for this clone.

## Methods

### Study design and study population

This was a retrospective case – case study. All primary MRSA cases with *spa* types t002, t008, t127, t304, t223, and t4549 with a sample date in 2022 or 2023 were extracted from the Danish national surveillance system on 16 November 2023. The study period was selected to ensure participants could reliably recall relevant exposure histories. Cases identified through screening were excluded. All MRSA cases with *spa* type t4549 were considered cases, while MRSA cases with *spa* types t002, t008, t127, t304, and t223 were considered case-*controls* (hereafter referred to as controls). These control *spa* types were selected based on their prevalence [7], commonality, and being typical MRSA *spa* types without known exposure. MRSA cases and controls that had died or moved away since their sample was taken were excluded from this study. Controls were randomly 1:2 frequency matched to cases based on age group and sex.

### Data collection

A questionnaire was developed to capture information on living environment, animal contact, and free time activities. This questionnaire was pilot tested through nine structured phone interviews with MRSA t4549 cases. These cases were excluded from the main study. Following the pilot interviews, adjustments to the questionnaire were made to improve the wording and reduce the number of questions.

Two versions of the final questionnaire were prepared, one for adults and one for children (<15 years). Slight variations between these two questionnaire versions existed to reflect differences in activity due to age (e.g. adults being asked about their profession). To limit recall bias, the maximum time period for past events asked about was 6 months prior to the sample date.

The questionnaires were prepared in SurveyXact [8] and distributed with a personalized invitation letter including a link to the survey and information about the use of the participant’s personal data. The letter was sent through e-Boks, a digital mailbox system used in Denmark for electronic communications for everyone over 15 years of age, linked to each individual’s personal registration number, a unique identifier that all residents in Denmark have [9]. Participants provided their informed consent by completing the electronic questionnaire. For children under the age of 15 years, the questionnaire was sent to their parent(s) or guardian(s) with the same registered home address as the child. Three selected case controls under the age of 15 years had to be re-sampled as contact details of the parental or guardians were not available.

The final questionnaires can be found in Supplementary Material S1. The questionnaires were sent out on 2 February 2024; up to two reminders were sent upon no reply. The data collection ended on 15 May 2024.

### Data analysis

Descriptive analyses were performed on demographic and clinical characteristics. Response rates were calculated, and age and sex distribution was compared between responders and non-responders using a chi2/Fisher’s exact test or t-test. A logistic regression analysis was performed to identify factors associated with MRSA t4549 infections. Matching variables were not included in the analysis. In a first step, a univariable analysis was performed for all individual variables that were gathered from both children and adults. Those that had less than five observations in both study groups were excluded from the analysis. All variables included in the univariable analysis that showed a model fit superior to the intercept-only model (i.e. a model including zero predictor variables), as indicated by a lower Akaike Information Criterion (AIC), were included in a multivariable stepwise forward logistic regression analysis. Variables were added to the model one at a time using the model selection-oriented *steps()* function of the “stats” package in R [10]. This function uses a stepwise variable selection procedure based on the AIC. It selects the variable with the best model fit and then repeats the process, adding the remaining variables one at a time. Multicollinearity between the variables in the final model was tested using the *Variance Inflation Factor.* The fit of the final model was determined using the Hosmer and Lemeshow Goodness-of-Fit Test. A p-value below 0.05 was considered significant.

All statistical analyses were conducted using R Statistical Software version 4.3.1.

## Results

### Demographic and clinical characteristics of cases and case-controls

A total of 121 MRSA t4549 cases and 242 controls (71 with *spa* type t304, 52 with t008, 49 with t127, 38 with t002, and 32 with t223) were invited to this study and asked to complete the questionnaire, of which 53% of cases (64/121) and 43% of controls (103/242) responded. Seventy-five percent of cases were male, and 17% were children under 15 years (Table 1). Respondents were distributed across the country. The most commonly reported site of infection in both cases and controls was the skin, although this was more commonly reported in cases (78% vs. 63%). Among those with skin infections, significantly more cases experienced infections on the foot (28% vs. 8%; p = 0.004) and toe (54% vs. 6%; p < 0.001) compared to controls.

**Table 1.** Demographic and clinical characteristics of cases (N = 64) and controls (N = 103)

	Cases (N = 64)	Controls (N = 103)
	N (%)	N (%)
Age group		
0–17	16 (25)	18 (18)
18–24	2 (3)	7 (7)
25–44	12 (19)	23 (22)
45–64	19 (30)	30 (29)
65+	15 (23)	25 (24)
Sex		
Female	16 (25)	25 (24)
Male	48 (75)	78 (76)
Adult/child		
Adult	53 (83)	85 (83)
Child	11 (17)	18 (18)
Travel		
Yes	22 (36)	29 (30)
No	31 (51)	57 (59)
Unknown	8 (13)	10 (10)
Regions		
Capital Region	18 (28)	41 (40)
Central Denmark	16 (25)	15 (15)
North Denmark	6 (9)	5 (5)
Southern Denmark	10 (16)	26 (25)
Zealand	14 (22)	15 (15)
Unknown	0 (0)	1 (1)

### Key determinants and associations of MRSA t4549 infection

Results for the individual variables are summarized in Table 2. The most common animal contacts reported by cases in the 6 months before the sample being taken were dogs (60% vs. 43% in controls), cats (23% vs. 26% in controls), and pheasants (7.8% vs. 1.0% in controls). Both contact with dogs as well as contact with pheasants were significantly associated with being an MRSA t4549 case in the univariable model, while contact with pheasants was also included in the multivariable model and remained significant (Table 2). Cases were nearly nine times more likely to have reported contact to pheasants compared to controls, though the confidence intervals being wide (OR = 8.6; 95%CI 1.4–167.7).

**Table 2.** Percentage frequency distribution and logistic regression analysis results for being a MRSA t4549 case

Variable	Cases (N = 64)	Controls (N = 103)	Univariable analysis (OR (95% CI))	Multivariable analysis (OR (95% CI))
Camping	4(6%)	7 (7%)	0.91 (0.23–3.16)	
Gym	16 (25%)	24 (23%)	1.1 (0.52–2.26)	
Playing indoor team sports	7 (11%)	2 (2%)	6.2 (1.44–42.57)	7.54 (1.58–54.82)
Jogging	24 (38%)	28 (27%)	1.61 (0.82–3.14)	
Playing outdoor team sports	12 (19%)	8 (8%)	2.74 (1.07–7.4)	
Visiting a sauna	11 (17%)	9 (9%)	2.17 (0.84–5.7)	
Swimming	27 (42%)	18 (18%)	3.45 (1.71–7.11)	4.15 (1.97–9.03)
Doing water sports	5 (8%)	2 (2%)	4.28 (0.89–30.54)	4.71 (0.88–35.78)
Contact with cat	15 (23%)	27 (26%)	0.86 (0.41–1.76)	
Contact with dog	39 (61%)	44 (43%)	2.09 (1.11–3.99)	1.83 (0.91–3.74)
Contact with pheasant	5 (8%)	1 (1%)	8.64 (1.35–167.73)	8.70 (1.25–174.29)
Living in apartment	14 (22%)	43 (42%)	0.39 (0.19–0.78)	
Living in townhouse	40 (63%)	41 (40%)	2.52 (1.34–4.84)	
Travel	22 (34%)	29 (28%)	1.34 (0.68–2.61)	

*Note:* Variables with fewer than 5 observations for both cases and controls, as well as variables not included in the survey questionnaire for all participants, were excluded from the regression analyses.

Different kinds of sports were reported by cases, who generally reported sports activities more frequently than controls: swimming (42% vs. 18%), jogging (38% vs. 27%), gym (25% vs. 23%), playing outdoor team sports (19% vs. 8%), indoor team sports (11% vs. 2%), and doing water sports (8% vs. 2%) (Table 2). Out of all of these sports activities, both indoor team sports and swimming significantly increased the odds of being a case in both the univariable and multivariable regression analyses. Cases had seven times higher odds of having reported indoor team sports compared to controls (OR = 7.54, 95%CI: 1.58–54.82), while the odds of being a case were four times higher for swimming (OR = 4.15, 95%CI: 1.97–9.03) (Table 2). Almost all cases who reported swimming had been swimming in a swimming pool (25/27, 92.6%), while nearly half had been swimming in the sea (12/27, 44%). Outdoor team sports was significantly associated with being a case in the univariable model (OR = 2.74, 95%CI: 1.07–7.4) but did not reach the multivariable regression analysis stage.

A slightly higher proportion of cases than controls reported a travel history (34% vs. 28%), but this difference was not statistically significant. In addition, more cases than controls reported having lived in a townhouse (63% vs. 40%). This was also significantly associated with being a case in the univariable regression analysis (OR 2.52, 95%CI: 1.34–4.48), but it did not reach the final multivariable regression model. There was also a significant association between having access to a garden and being a case regardless of housing type (OR 3.39, 95%CI: 1.38–9.63).

There was little correlation (VIF = 1.03–1.04) between the predictors in our final regression model, and the Hosmer and Lemeshow Goodness-of-Fit test indicated a good model fit (p = 0.6601).

## Discussion

To the best of our knowledge, this is the first national study exploring potential factors associated with the emerging MRSA t4549, a *spa* type that distinguishes itself from other MRSA types without known exposure. The vast majority of cases were male, and significantly more cases reported having had the infection on toes or feet.

In the multivariable regression model, contact with pheasants was significantly more common in cases than in controls. Although only a few cases could be explained by exposure to pheasants, with low case numbers and large confidence intervals, pheasants and other wild birds have been shown to be carriers of foodborne pathogens, and *S. aureus* has been reported from wild birds, including pheasants [11–13]. It is possible that pheasants are a proxy for contact with wild birds in general.

Cases had significantly higher odds to have reported indoor team sports and swimming. While there are no other studies reporting on risk factors of this *spa* type or this sequence type in specific, swimming has been found to be a risk factor for other community-associated MRSA (CA-MRSA) types [14–16]. Swimming in swimming pools and other recreational water facilities can spread MRSA through direct and indirect contact with infected individuals. Asymptomatic carriers can contaminate water, transforming it into a transient reservoir for MRSA, especially when not properly chlorinated. This poses a risk of colonization to new hosts, particularly those with open wounds [14–16]. Recreational water use, especially swimming, is a common activity, particularly for children and young people, which aligns with findings for other CA-MRSA strains, where young people are more frequently affected, although it does not explain why more males were affected [14].

Our study found some evidence that playing indoor team sports might be linked to MRSA t4549 transmission. Although we did not ask about the type of indoor sports, this finding is consistent with existing literature suggesting that athletes, particularly those involved in competitive contact sports, are at increased risk of CA-MRSA infection [17]. Physical contact, shared facilities and equipment, and hygiene practices contribute significantly to the transmission of MRSA among sports participants [18]. Indirect contact through contaminated items such as towels, razors, and locker room surfaces also contributes to the spread of MRSA, regardless of proper chlorine levels, especially when it contacts uncovered cuts or scrapes [15, 17]. Sports-associated skin damage, such as abrasions and lacerations, increases the risk of MRSA infection by disrupting the skin’s barrier, facilitating bacterial entry. Sharing sports equipment also plays a significant role in bacterial transmission [17].

Proper cleaning of training environments is essential to prevent the spread of MRSA, as *S. aureus* can survive on surfaces [19]. Preventing CA-MRSA infections among sport participants involves personal, environmental, and healthcare measures. Sport participants should practice good hygiene, such as washing hands regularly, showering after activities, and not sharing personal items. Facilities and equipment should be thoroughly cleaned and disinfected. Educating athletes and their coaches about the risks of MRSA and timely treatment of skin infections is also critical.

Further studies are needed to confirm our current findings and to better delineate the role of the environment and wildlife in relation to MRSA t4549. The molecular composition of MRSA t4549 may suggest an environmental reservoir such as an animal host. Phylogenetic studies show that some animal-adapted staphylococci are derived from human strains and can now be transmitted back to humans [20]. Wild animals interacting with diverse environmental microbiomes may carry new strains and act as vehicles for their transmission to humans. New animal MRSA strains can introduce novel resistance determinants and virulence factors into humans [21, 22]. We found significantly more cases involving contact with pheasants compared to the controls, albeit with small case numbers. The occurrence of CA-MRSA has previously been associated with wild birds [23], and future research should investigate this interesting finding, considering the potential role of pheasants in MRSA transmission. In addition, research should focus on developing effective educational programs for swimmers and other sport participants to minimize transmission by reducing the sharing of equipment and facilities and encouraging showering after exercise. These efforts will contribute to more comprehensive prevention strategies and a deeper understanding of MRSA dynamics in different settings.

## Methodological considerations

To our knowledge, this is the first case – case study to identify factors associated with MRSA t4549 infections and provides a unique insight into this *spa* type. We included all recent MRSA t4549 cases in this study, increasing the reliability of the findings. Despite Denmark’s low MRSA prevalence, the sample sizes of both cases and controls allowed for the detection of odds ratios of 2.4 with 80% power at an alpha of 0.05. However, it is possible that factors with weaker associations may have been underpowered to detect. A case – case study design was chosen over a case – control investigation because it allows for the examination of differences among individuals who have all been diagnosed with MRSA, thereby controlling for biases associated with being diagnosed with the infection itself. However, the study also faces limitations. The variables included are relatively unspecific, which may limit the specificity of the findings. In addition, due to the low prevalence of MRSA in Denmark, the number of cases of MRSA t4549 was inherently low, and the results must be interpreted with caution. Despite efforts to identify potential points of transmission, the precise source of infections in the community remains uncertain and warrants further investigation. The limited international knowledge of this *spa* type may limit the generalizability of the findings. Finally, the widespread distribution of MRSA t4549 underscores the need to identify the environmental source but also complicates efforts to identify its exact source and presents a challenge for future research efforts.

## Conclusions

To our knowledge, this is the first analytical epidemiological study to focus on this *spa* type. Pheasant exposure, indoor team sports, and swimming emerged as notable factors despite the low case numbers. Although the results, due to the small number of cases, should be interpreted with caution, it is important to emphasize the potential implementation of preventive measures at both the individual and sports facility levels. The emerging nature and increasing prevalence of this *spa* type in Denmark underline the importance of identifying public health measures to effectively reduce the risk of MRSA transmission.

## Supporting information

Young et al. supplementary material Young et al. supplementary material

## Data Availability

The data supporting the findings of this study are not publicly available due to confidentiality agreements and ethical restrictions. However, de-identified data may be made available upon reasonable request, subject to relevant approvals.

## Supplementary material

For supplementary material accompanying this paper visit http://doi.org/10.1017/S0950268825000299. click here to view supplementary material
