# Supplementary material for: First nationwide case – case study on factors associated with emerging methicillin-resistant Staphylococcus aureus spa type t4549 in Denmark, 2022–2023
Source: Epidemiol Infect. 2025 Mar 12;153:e52. doi: 10.1017/S0950268825000299 (PMC12001147; doi:10.1017/S0950268825000299)
Supplement: Young et al. supplementary material [file S0950268825000299sup001.pdf]

SUPPLEMENTARY FILES

Questionnaire 1 – sent to all participants over 15 years of age

[This questionnaire was originally sent out in Danish and has been translated for the purposes of this manuscript]

Welcome to the study on risk factors for MRSA infection.

In *[samplemonth]* you were diagnosed with MRSA. We would like to ask you some questions about your MRSA infection and how it happened. Your answers will help us to understand the risk factors for MRSA infection.

It will take between 10 and 15 minutes to complete the questionnaire.

Where on the body were you infected (you can tick more than one place)?

- o Skin (e.g. wounds)
- o Ears
- o Eyes
- o Upper respiratory tract (e.g. sore throat, sinusitis, otitis media or sores in the nose)
- o Lower respiratory tract (e.g. pneumonia or bronchitis)
- o Bones or joints
- o In the blood (e.g. blood poisoning, bacteremia or sepsis)
- o The brain or spinal cord (e.g. cerebrospinal fluid)
- o Urinary tract
- o Other place:
- o Don't remember / don't know

Any comments about the site of infection:

____

You indicated that you had MRSA on your skin. Where on your skin were you infected (you can tick more than one place)?

- o Toes or toenails
- o Feet
- o Lower leg
- o Thigh
- o Stomach or back
- o Arms

- Hands or fingernails
- Face
- Other place:

Any comments:

____

Have you had any wounds or other skin problems (e.g. eczema) in the 6 months before you were diagnosed with MRSA?

- Yes
- No
- Don't remember / don't know

Please provide more details about the wound or your skin problem(s):

____

Do you have any idea what or how you became infected?

- Yes
- No

Please describe how you think you were infected:

____

How many people lived in your household when you were diagnosed with MRSA? (including yourself).

Number of people:

____

Were others in your household also examined for MRSA in connection with you being diagnosed with MRSA?

- Yes
- No
- Don't remember / don't know

What was the result of the examination of your household member(s)?

- One or more household members tested positive
- All household members tested negative
- Don't remember / don't know

Any comments:

____

Did you have a tattoo in the 6 months before you were diagnosed with MRSA?

- Yes
- No, but I have older tattoos
- No, I have no tattoos
- Does not want to answer

Have you had any of the following illnesses or health problems in the 6 months before you were diagnosed with MRSA (you can tick more than one box)?

- No
- Asthma (incl. allergic asthma)
- Allergies (not allergic asthma)
- High blood pressure
- Diabetes / diabetes
- Cardiovascular disease
- Chronic bronchitis, chronic obstructive pulmonary disease (COPD), oversized lungs (pulmonary emphysema).
- Kidney disorders
- Cancer
- Mental health problems (e.g. depression, anxiety)
- Overweight
- Autoimmune diseases (e.g. rheumatoid arthritis, lupus)
- Brain and nerve disease (e.g. epilepsy, multiple sclerosis)
- Thyroid disease
- Stomach and intestinal disorders (e.g. irritable bowel syndrome)
- Other: _____

What type of accommodation have you lived in in the last 6 months before you were diagnosed with MRSA (you can tick more than one box)?

- Farm or disused agricultural property
- Detached house or terraced house
- Apartment
- Nursing home or other institution
- Other: _____
- Don't remember / don't know

Did you have access to a garden in the 6 months before you were diagnosed with MRSA?

- Yes
- No
- Don't remember / don't know

What did you do in the 6 months before you were diagnosed with MRSA (you can tick more than one box)?

- Has a job
- Goes to school / is a student
- Not in employment (e.g. pensioner or late pensioner)
- Are homemakers
- Other: _____
- Does not wish to disclose

We would like to know in which industry you worked in the last 6 months before you were diagnosed with MRSA (you can tick more than one box)?

- Building and construction
- Finance and insurance
- Trade and service
- Hotel and restaurant
- Agriculture and forestry
- Health and welfare
- Teaching and research
- Other: _____
- Don't know / don't want to answer

What leisure activities did you do in the 6 months before you were diagnosed with MRSA (you can tick more than one box)?

- o Training in the gym (incl. team training)
- o Outdoor crossfit or similar
- o Football
- o Other outdoor team sports (e.g. American football or rugby)
- o Indoor team sports (e.g. handball, volleyball or basketball)
- o Water sports (eg sailing, water skiing, rowing, canoeing or kayaking)?
- o Martial arts (e.g. wrestling, karate or taekwondo)
- o Swimming (incl. bathing)
- o Sauna or similar
- o Camping
- o Fishing
- o Hunting
- o Running / jogging
- o Walk in nature
- o Others: _____
- o None of the above

Have you swum in any of these places in the 6 months before you were diagnosed with MRSA (you can tick more than one)?

- o Swimming pool
- o Lake
- o Sea
- o River
- o Own swimming pool
- o Other:
- o None of the above

Have you had contact with domestic animals or pets within the last 6 months before you were diagnosed with MRSA (you are welcome to tick several places)?

- o Dog
- o Cat
- o Rodents (e.g. mice, rats, chinchillas, hamsters or guinea pigs)
- o Reptiles (e.g. turtles or snakes)
- o Pig
- o Cattle
- o Sheep
- o Goat

- Poultry
- Horse
- Other animals: _____
- No contact with livestock or pets

Have you been in contact with other animals at home, in your garden or out in the wild within the last 6 months before you were diagnosed with MRSA (you are welcome to tick several places)?

- Mouse
- Rat
- Hedgehog
- Pheasant
- Hare
- Squirrel
- And
- Goose
- Fox
- Deer
- Other animals: _____
- No contact with other animals

Have you been abroad within the last 6 months before you were diagnosed with MRSA?

- Yes
- No
- Don't remember / don't know

Which country(s) have you traveled to within the last 6 months before you were diagnosed with MRSA? You can specify up to five countries.

[displays a list of countries that can be selected]

The last thing we would like to ask you is if we can contact you again if we have more questions?

- Yes
- No

If you have any additional comments, please enter them here:

____

This is the end of the survey. Thank you very much for your participation.

If you click on "EXIT", the questionnaire will be sent.

Questionnaire 2 – sent to all parents of participants under 15 years of age

[This questionnaire was originally sent out in Danish and has been translated for the purposes of this manuscript]

Welcome to the study on risk factors for MRSA infection.

In [*sample month*], your child [*first name*] [*last name*] was found to have MRSA. We would like to ask you some questions about your child's MRSA infection and what happened before it. Your answers will help us to better understand the risk factors for MRSA infection.

It will take between 10 and 15 minutes to complete the questionnaire.

Who is completing the questionnaire?

- o Mother
- o Father
- o Guardian
- o Other: _____

Where on the body was your child infected (you can tick more than one place)?

- o Skin (e.g. wounds)
- o Ears
- o Eyes
- o Upper respiratory tract (e.g. sore throat, sinusitis, otitis media or sores in the nose)
- o Lower respiratory tract (e.g. pneumonia or bronchitis)
- o Bones or joints
- o In the blood (e.g. blood poisoning, bacteremia or sepsis)
- o The brain or spinal cord (e.g. cerebrospinal fluid)
- o Urinary tract
- o Other place:
- o Don't remember / don't know

Any comments about the site of infection:

____

You indicated that your child had MRSA on the skin. Where on the skin was your child infected (you can tick more than one place)?

- o Toes or toenails
- o Feet
- o Lower leg
- o Thigh
- o Stomach or back
- o Arms
- o Hands or fingernails
- o Face
- o Other place:

Any comments:

____

Has your child had any wounds or other skin problems (e.g. eczema) in the 6 months before you were diagnosed with MRSA?

- o Yes
- o No
- o Don't remember / don't know

Please provide more details about your child's wound or skin problem(s):

____

Do you or your child have any idea what or how your child became infected?

- o Yes
- o No

Please describe how you think your child was infected:

____

How many people lived in your child's household when MRSA was detected? (including your child themselves).

Number of people:

_____

Were others in your child's household also examined for MRSA in connection with your child being diagnosed with MRSA?

- o Yes
- o No
- o Don't remember / don't know

What was the result of the survey of the household members?

- o One or more household members tested positive
- o All household members tested negative
- o Don't remember / don't know

Any comments:

____

Has your child had any of the following illnesses or health problems in the 6 months before you were diagnosed with MRSA (you can tick more than one box)?

- o No
- o Asthma (incl. allergic asthma)
- o Allergies (not allergic asthma)
- o High blood pressure
- o Diabetes / diabetes
- o Cardiovascular disease
- o Chronic bronchitis, chronic obstructive pulmonary disease (COPD), oversized lungs (pulmonary emphysema).
- o Kidney disorders
- o Cancer
- o Mental health problems (e.g. depression, anxiety)
- o Overweight
- o Autoimmune diseases (e.g. rheumatoid arthritis, lupus)
- o Brain and nerve disease (e.g. epilepsy, multiple sclerosis)
- o Thyroid disease
- o Stomach and intestinal disorders (e.g. irritable bowel syndrome)
- o Other: _____

What type of accommodation has your child lived in in the last 6 months before they were diagnosed with MRSA (you can tick more than one box)?

- o Farm or disused agricultural property
- o Detached house or terraced house
- o Apartment
- o Nursing home or other institution
- o Other: _____
- o Don't remember / don't know

Has your child had access to a garden in the 6 months before they were diagnosed with MRSA?

- o Yes
- o No
- o Don't remember / don't know

Did your child attended an institution in the 6 months before they were diagnosed with MRSA (you can tick more than one box)?

- o No, the child has not attended an institution
- o Day nursery or crèche
- o Regular nursery school
- o Forest kindergarten
- o School
- o Other: _____

Did your child have a part-time job within the last 6 months before MRSA was found?

- o Yes
- o No
- o Don't remember / don't know

Please describe the part-time job(s):

____

What leisure activities did your child do in the last 6 months before MRSA was found (you can tick more than one box)?

- Played in a garden
- Played in a playground
- Training in the gym (incl. team training)
- Outdoor crossfit or similar
- Football
- Other outdoor team sports (e.g. American football or rugby)
- Indoor team sports (e.g. handball, volleyball or basketball)
- Water sports (eg sailing, water skiing, rowing, canoeing or kayaking)?
- Martial arts (e.g. wrestling, karate or taekwondo)
- Swimming or bathing (including sweeping)
- Sauna or similar
- Camping
- Fishing
- Hunting
- Running / jogging
- Walk in nature
- Others: _____
- None of the above

Did your child swim, bathe or sponge in any of these places in the 6 months before your child was diagnosed with MRSA (you can tick more than one place)?

- Swimming pool
- Lake
- Sea
- River
- Own swimming pool
- Other:
- None of the above

Has your child had any contact with pets or domestic animals in the 6 months before MRSA was diagnosed (you can tick more than one box)?

- Dog
- Cat
- Rodents (e.g. mice, rats, chinchillas, hamsters or guinea pigs)
- Reptiles (e.g. turtles or snakes)
- Pig
- Cattle

- Sheep
- Goat
- Poultry
- Horse
- Other animals: _____
- No contact with livestock or pets

Has your child been in contact with other animals at home, in the garden or outdoors in the 6 months before MRSA was found (you can tick more than one box)?

- Mouse
- Rat
- Hedgehog
- Pheasant
- Hare
- Squirrel
- And
- Goose
- Fox
- Deer
- Other animals: _____
- No contact with other animals

Has your child been abroad within the last 6 months before they were diagnosed with MRSA?

- Yes
- No
- Don't remember / don't know

Which country(s) has your child traveled to within the last 6 months before they were diagnosed with MRSA?
You can specify up to five countries.

[displays a list of countries that can be selected]

The last thing we would like to ask you is if we can contact you again if we have more questions?

- Yes
- No

If you have any additional comments, please enter them here:

____

This is the end of the questionnaire. Thank you very much for your participation.

When you press on "EXIT", will the schedule be sent .
